# Centrally adjudicated vs. investigator-reported outcomes in randomized heart failure trials

**DOI:** 10.1093/eurheartj/ehae753

**Published:** 2024-11-09

**Authors:** Simon Wandel, Akshay S Desai, Chien-Wei Chen, John J V McMurray, Milton Packer, Scott D Solomon, Marc A Pfeffer, G Michael Felker, Faiez Zannad, Mark C Petrie, Pardeep S Jhund, Zenab Attari, Guenther Mueller-Velten, Martin Lefkowitz, David Soergel, Claudio Gimpelewicz

**Affiliations:** Development, Novartis Pharma AG, Forum 1, Novartis Campus, CH-4056 Basel, Switzerland; Cardiovascular Division, Brigham and Women’s Hospital, Harvard Medical School, Boston, MA, USA; Novartis Pharmaceuticals Corporation, East Hannover, NJ, USA; British Heart Foundation Cardiovascular Research Centre, University of Glasgow, Glasgow, UK; Baylor Heart and Vascular Institute, Baylor University Medical Center, Dallas, TX, USA; Imperial College, London, UK; Cardiovascular Division, Brigham and Women’s Hospital, Harvard Medical School, Boston, MA, USA; Cardiovascular Division, Brigham and Women’s Hospital, Harvard Medical School, Boston, MA, USA; Division of Cardiology, Duke University School of Medicine and Duke Clinical Research Institute, Durham, USA; Université de Lorraine INSERM, Centre d’Investigations Cliniques Plurithématique 1433, INSERM U1116, CHRU de Nancy, F-CRIN INI-CRCT, Nancy, France; British Heart Foundation Cardiovascular Research Centre, University of Glasgow, Glasgow, UK; British Heart Foundation Cardiovascular Research Centre, University of Glasgow, Glasgow, UK; Novartis Healthcare Pvt. Ltd, Hyderabad, India; Development, Novartis Pharma AG, Forum 1, Novartis Campus, CH-4056 Basel, Switzerland; Novartis Pharmaceuticals Corporation, East Hannover, NJ, USA; Development, Novartis Pharma AG, Forum 1, Novartis Campus, CH-4056 Basel, Switzerland; Development, Novartis Pharma AG, Forum 1, Novartis Campus, CH-4056 Basel, Switzerland

**Keywords:** Adjudication, Investigator-reported outcomes, Heart failure, Cardiovascular trial

## Abstract

**Background and Aims:**

Heart failure endpoints in cardiovascular outcome trials are commonly identified through centralized adjudication of investigator-reported events. It remains unclear whether central adjudication improves the accuracy of treatment effect estimates in terms of log[hazard ratios (HR)].

**Methods:**

Data from seven cardiovascular outcome trials with >1000 patients that included centrally adjudicated heart failure outcomes were utilized to assess (i) the concordance between investigator-reported and centrally adjudicated heart failure and cardiovascular death events; (ii) rates of subsequent all-cause mortality following positively vs. negatively adjudicated heart failure events; and (iii) the correlation of log(HR) based on centrally adjudicated vs. investigator-reported events.

**Results:**

Positive adjudication rates for investigator-reported events varied widely across trials, but were generally higher for cardiovascular death (range: 87.9%–99.2%) than for heart failure hospitalization (range: 61.6%–88.0%). The risk for subsequent all-cause death was similar for positively and negatively adjudicated heart failure hospitalizations. Log(HR) correlated well for cardiovascular death [*R*^2^ = .80, 95% credible interval (CrI): 0.53–0.93] and the composite of cardiovascular death or heart failure hospitalization (*R*^2^ = .79, 95% CrI: 0.46–0.93), but less for heart failure hospitalization (*R*^2^ = .57, 95% CrI: 0.10–0.83).

**Conclusions:**

Positive adjudication rates were lower for heart failure events than cardiovascular death, but even negatively adjudicated heart failure events are prognostically important. Central adjudication of events did not alter the results (precision or estimated log(HR)), though some variation was observed, depending on the indication. The results suggest that the decision to pursue centralized adjudication of heart failure events in a specific trial may need to be individualized.

## Structured Graphical Abstract

**Structured Graphical Abstract:**
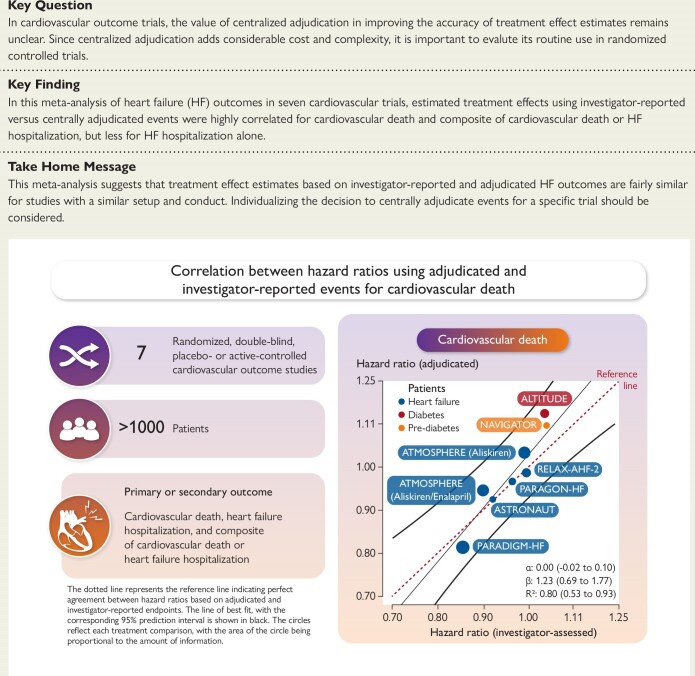
The graphical abstract figure shows the results of 7 studies for which investigator-reported and adjudicated heart failure outcomes were available, illustrates the agreement of respective log hazard ratios for cardiovascular death, and summarizes the key findings.

## Introduction

Heart failure (HF) outcomes reported by investigators in cardiovascular (CV) outcome trials are commonly referred for centralized adjudication by a blinded committee of clinical experts to select relevant clinical endpoints for analysis.^1^ This process has been encouraged by regulators on the premise that standardization of endpoint ascertainment through centralized review will both enhance the face validity of trial results and reduce bias by filtering the ‘noise’ of spurious events that might be insensitive to the effects of the treatment under study.^2^ The value of centralized adjudication is generally felt to be greater in studies utilizing composite endpoints that include non-fatal events, such as HF hospitalization (HFH) or acute coronary syndromes, in which local assessment by investigators may be more vulnerable to variations in clinical judgement or regional practice.^1^

Cardiovascular trials are typically expensive,^3^ and centralized adjudication of clinical endpoints adds considerable cost (estimated mean cost per event corrected by adjudication > £2000)^4^ and complexity to clinical trial operations. Therefore, there has been increasing debate regarding the need for routine deployment of this approach, particularly in randomized controlled trials that naturally mute the potential bias introduced by reliance on investigator-reported events.^1^ Indeed, recent data from HF trials suggest that treatment effect estimates in terms of log[hazard ratios (HR)] based on adjudicated HF outcomes do not vary substantially from those based on investigator-reported outcomes.^5,6^ Additionally, strict endpoint criteria may lead the clinical endpoints committee (CEC) to negatively adjudicate potentially relevant events merely due to source documentation limitations, rather than a strong conviction that the judgement of investigators is flawed.^7–10^

To better understand the incremental value of centralized adjudication of HF outcomes across a range of CV outcome trials spanning different patient populations, we conducted a participant-level meta-analysis of seven prospective, randomized trials from a single sponsor to assess (i) the concordance for investigator-reported HF and CV death events; (ii) the rates of subsequent all-cause mortality following positively adjudicated vs. negatively adjudicated HF events; and (iii) the variation in the log(HR) based on centrally adjudicated vs. investigator-reported events.

## Methods

### Study selection

We identified all Novartis-sponsored, completed randomized, double-blind, placebo- or active-controlled CV outcome studies from 1997 to 2019 in which a CEC was deployed for event adjudication and patients had provided written informed consent for the use of study data. Studies eligible for inclusion in this analysis were those with a sample size of >1000 patients (large trials) with both CV death and HFH as a (or as a component of a) pre-specified primary or secondary outcome and where data regarding both investigator-reported and centrally adjudicated events were available. In all studies, the CEC was blinded to treatment assignment when conducting the event adjudication. Given that this meta-analysis comprises studies funded by a single sponsor, certain elements of the PRISMA-IPD guidelines^11^ may not be fully applicable. Nonetheless, the PRISMA-IPD flow diagram is available in the Supplementary data online.

### Investigator-reported vs. centrally adjudicated events

Detailed criteria for classification of CV death and HFH are outlined in the CEC charters for each trial. While specific endpoint definitions varied across trials, most studies utilized definitions for CV death and HFH (see Supplementary data online, *Tables S1* and *S2*) that are substantially similar to those codified in the 2008 United States Food and Drug Administration (US FDA) guidance.^12^ All investigator-reported occurrences of death and HFH were centrally adjudicated in each of the selected trials. In each trial, for every investigator-reported clinical event (either spontaneously or at the behest of the sponsor), a documentation package was submitted to the CEC, where it was independently reviewed by two experts to assess whether the pre-defined criteria for CV death or HFH endpoint were met. Disagreements between reviewers were pushed to committee discussion for further review and consensus adjudication, with ties broken by the CEC chair. Events unreported by investigators but identified by the CEC independently during the adjudication process were also included in this analysis.

### Statistical methods

We analysed each of the seven selected trials to examine the concordance between investigator-reported and centrally adjudicated HF events and the prognosis associated with positively and negatively adjudicated HF events. Concordance was assessed using agreement rates (percentage of positively adjudicated events among all investigator-reported events), overall and by region. Furthermore, we employed meta-analytic techniques to pool data across the trials to assess the impact of adjudicated vs. unadjudicated event data on the log(HR). All analyses were performed using SAS Version 9.4 (SAS Institute, Cary, NC), R (≥3.4.3),^13^ and JAGS (≥4.3.0).^14^ Descriptive statistics [mean, standard deviation (SD); median, range; number of patients (*N*), percentage] were used to describe the study populations and the number and rates (per 100 patient-years) of first events. For inferential analyses, 95% confidence intervals (CI) were used, with the exception of the Bayesian model, for which 95% credible intervals (CrIs) for the intercept, slope, and *R*^2^ and prediction intervals (PI) for the figure are reported.

All analyses used the full analysis set, which included all randomized patients and followed the intention-to-treat principle. Cardiovascular death, HFH, and the composite of CV death or HFH were analysed as time to first occurrence of the event or the composite in Cox proportional hazards models. All individual participant-level data were anonymized in compliance with international data protection policies. A ±30-day window for event occurrence date was applied to decide whether an investigator-reported HFH was positively adjudicated, meaning that a CEC confirmed HFH occurred within this window. All other events were deemed negatively adjudicated.

At the trial level, the correlation between the log(HR) based on investigator-reported events and the log(HR) based on adjudication-confirmed events was investigated. Trial-specific log(HR) and corresponding standard error (SE) using Cox proportional hazards models were estimated first, with treatment as a covariate. The trial-specific log(HR) were meta-analysed with a Bayesian model using a linear relationship and an ‘error-in-variable’ approach. This model considered the correlation of log(HR) induced by multi-arm studies, which was obtained directly from the Cox model. Additionally, it was necessary to estimate the within-study correlation of log(HR) from investigator-reported and adjudication-confirmed events, for which 1000 bootstrap samples were used for each study.^15^ The model expresses log(HR) based on adjudication-confirmed events (the ‘dependent variable’) as a linear function of log(HR) based on investigator-reported events (the ‘independent variable’). Hereby, the intercept reflects a systematic difference (bias), and the slope a dependence on the size of the log(HR); in a perfect situation, the intercept would be 0 and the slope 1. To quantify the strength of the linear association, an inverse variance-weighted version of the Bayesian *R*^2^ statistic was calculated, which may be interpreted as an approximation of the squared correlation coefficient.^16^ To study the effect on the precision of the estimates, the information gain/loss was calculated, which we defined as 100 ∗ (1 − (se[log(HR_inv)])^2^/(se[log(HR_adj)])^2^. Here, positive/negative values imply a higher/smaller precision of the log(HR) when using investigator reported as opposed to adjudicated events. In a sensitivity analysis, Cox models with treatment by region interaction were used to explore the influence of region.

To study the prognostic value of first adjudication-confirmed HFH, as opposed to non-confirmed HFH, with regard to subsequent all-cause mortality, patients were classified into the following three groups: (i) adjudication-confirmed investigator-reported HFH; (ii) adjudication non-confirmed investigator-reported HFH; and (iii) no investigator-reported HFH. Patients who died on the day of HFH were included in group 3. Group membership was implemented as a time-varying covariate to avoid immortal time bias,^17^ which can lead to severe misinterpretation of the data and confound results. Both unadjusted and adjusted Cox models were fitted to obtain study-specific HRs for the subsequent all-cause mortality risk when comparing adjudication-confirmed, non-confirmed, and no investigator-reported HFH. Additional details of the statistical analyses, including JAGS code, can be found in the Supplementary data online.

## Results

### Characteristics of trials

We identified seven double-blind, randomized controlled trials including 46 691 patients with HF with reduced ejection fraction (HFrEF), HF with preserved ejection fraction (HFpEF), acute HF, and diabetes/pre-diabetes that had investigator-reported and CEC-adjudicated data regarding CV death and HFH (*Table 1*). Two trials for HFrEF,^18–21^ one for HFpEF,^22^ and two each for acute HF (AHF)^23,24^ and diabetes/pre-diabetes^25–27^ were included.

**Table 1:** Summary of study design, outcomes, treatments, and populations

Study name (follow-up duration)	Study design	Primary endpoint	Key secondary or exploratory endpoints^a^	Population (randomized set)	Key results
HFrEF and HFpEF					
PARADIGM-HF^18,20^ (median = 27 months)	Double-blind, parallel group, active-controlled trial Superiority of sacubitril/valsartan vs. enalapril	Composite of CV death or first HFH	EE: first occurrence of a composite of CV death, HFH, NMI, NFS, or resuscitated sudden death	*n* = 8442 Chronic HF, NYHA II–IV, EF ≤ 35%	Sacubitril/valsartan was superior to enalapril in reducing the risk of death and HFH (*P* < .001)
ATMOSPHERE^19,21^ (median = 36.6 months)	Double-blind, three arm, active-controlled trial Superiority of aliskiren/enalapril combination vs. enalapril and non-inferiority of aliskiren vs. enalapril	Composite of CV death or first HFH	EE: ACM, CV death, HFH; CV composite endpoint (CV death, HFH, NMI, NFS, or resuscitated sudden death)	*n* = 7064 Chronic HF, NYHA II–IV EF ≤ 35%	No statistically significant difference in CV death or first HFH between treatments
PARAGON-HF^22^ (median = 35 months)	Double-blind, parallel group, active-controlled trial Superiority of sacubitril/valsartan vs. valsartan	Composite endpoint of CV death and total (first and recurrent) HFH	KS: KCCQ, NYHA, all-cause death, composite of renal death, ESRD, and ≥50% decline in eGFR	*n* = 4822 Chronic HF, NYHA II–IV, EF ≥ 45%	Sacubitril/valsartan was not superior to valsartan in reducing the rate of the composite endpoint of CV death and total (first and recurrent) HFH
Acute HF (HFrEF and HFpEF)					
ASTRONAUT^23^ (median = 11.3 months)	Double-blind, placebo-controlled trial Aliskiren vs. placebo	First occurrence of CV death or rehospitalization for HF at 6 months	KS: composite of CV death or worsening HF at 12 months First CV event (CV death, HFH, NMI, NFS, and resuscitated sudden death) at 12 months	*n* = 1639 Chronic HF with LVEF ≤ 40%	No difference in CV death or HF rehospitalization after 6 months and 12 months between aliskiren and placebo
RELAX-AHF-2^24^ (mean (±SD) = 167.6 ± 38.2 days for serelaxin group and 166.5 ± 40.0 days for placebo group)	Double-blind, placebo-controlled, event-driven trial Serelaxin vs. placebo	CV death at 180 days and worsening HF at 5 days	KS: composite of CV death or rehospitalization for HF or RF at 180 days	*n* = 6600 Hospitalized for acute HF with dyspnoea	No difference in incidence of CV death at 180 days or worsening HF at 5 days between serelaxin and placebo
Diabetes and pre-diabetes					
ALTITUDE^25^ (median = 32.9 months)	Double-blind, placebo-controlled trial Aliskiren vs. placebo	Composite of CV death, resuscitated cardiac arrest, NMI or FMI, NFS or FS, HFH, ESRD, death due to RF or loss of kidney function	KS: composite of CV death, resuscitated sudden death, NMI, NFS, HFH	*n* = 8606 Patients with T2DM and renal disease or CV disease	No difference in CV or renal outcomes between aliskiren and placebo
NAVIGATOR^26,27^ (median = 5.0 years for incident diabetes and median = 6.5 years for vital status)	Double-blind, placebo-controlled, 2 × 2 factorial design trial Valsartan/matching placebo vs. nateglinide/matching placebo (non-valsartan)	Incidence of DM; extended CV outcome—CV death, NMI, NFS, revascularization procedure, HFH, or unstable angina; core CV outcome—CV death, NMI, NFS or HFH	EE: time to death from all causes and the time to CV-related hospitalization	*n* = 9518 Patients with impaired glucose tolerance and CV risk factors or known CV disease	No difference in rate of CV outcomes in valsartan vs. placebo and nateglinide vs. placebo

ACM, all-cause mortality; CV, cardiovascular; DM, diabetes mellitus; EE, exploratory endpoints; EF, ejection fraction; eGFR, estimated glomerular filtration rate; ESRD, end-stage renal disease; FMI, fatal MI; FHF, fatal heart failure; FS, fatal stroke; HF, heart failure; HFH, heart failure hospitalization; HFpEF, heart failure with preserved ejection fraction; HFrEF, heart failure with reduced ejection fraction; IV, intravenous; KCCQ, Kansas City cardiomyopathy questionnaire; KS, key secondary; LVEF, left ventricular ejection fraction; MI, myocardial infarction; NFS, non-fatal stroke; NMI, non-fatal MI; NYHA, New York Heart Association; RF, renal failure; T2DM, type 2 DM; vs., versus.

^a^Only key endpoints relevant for present analyses are indicated.

### Individual concordance between investigator-reported and clinical endpoints committee-adjudicated clinical events

The positive adjudication rates for investigator-reported outcomes varied by trial and event type (*Table 2*). For CV death, rates ranged from 87.9% (NAVIGATOR^26,27^) to 99.2% (ASTRONAUT^23^). For HFH, rates ranged from 61.6% (ALTITUDE^25^) to 88.0% (RELAX-AHF-2^24^). Agreement rates for CV death were similar across regions, yet for HFH, Latin America seemed to have lower agreement rates than other regions (see Supplementary data online, *Table S3*).

**Table 2:** Agreement rates between investigator-reported and clinical endpoints committee-adjudicated events

Study	CV death	HFH
PARADIGM-HF^18,20^	1093 (96.6)	1090 (68.0)
ATMOSPHERE^19,21^	1285 (96.8)	1128 (62.2)
PARAGON-HF^22^	274 (90.7)	737 (69.2)
ASTRONAUT^23^	249 (99.2)	407 (80.0)
RELAX-AHF-2^24^	501 (92.8)	1072 (88.0)
ALTITUDE^25^	479 (90.9)	419 (61.6)
NAVIGATOR^26,27^	211 (87.9)	164 (72.9)

Agreement (%) was defined as 100 ∗ YY/(YY + YN).

CV, cardiovascular; HFH, heart failure hospitalization; YY, investigator-reported and positively confirmed; YN, investigator-reported, not confirmed.

### Prognostic value of an adjudication-confirmed vs. non-confirmed investigator-reported heart failure hospitalization on mortality

The adjudication decision was examined for whether an investigator-reported HFH influenced subsequent prognosis. The association between adjudication-confirmed vs. adjudication non-confirmed HFH, and subsequent all-cause mortality, was investigated. An investigator-reported HFH, whether confirmed by adjudication or not, was associated with a substantially higher risk of all-cause mortality, and this risk was similar for adjudication-confirmed and non-confirmed HFH (*Table 3*).

**Table 3:** Prognostic analysis of all-cause death after first heart failure hospitalization event

Study name	First HFH adjudication status		No HFH^a^	Unadjusted model		Adjusted model	
	Confirmed	Rejected		Confirmed vs. no HFH^a^	Not confirmed vs. no HFH^a^	Confirmed vs. no HFH^a^	Not confirmed vs. no HFH^a^
	All-cause death *n*/*N* (%), *n*/*T*, EAR (95% CI)	All-cause death *n*/*N* (%), *n*/*T*, EAR (95% CI)	All-cause death *n*/*N* (%), *n*/*T*, EAR (95% CI)	HR (95% CI)	HR (95% CI)	HR (95% CI)	HR (95% CI)
PARADIGM-HF	400/1089 (36.7%) 400/23.4 17.1 (15.5–18.9)	182/500 (36.4%) 182/11.0 16.6 (14.3–19.2)	964/6810 (14.2%) 964/151.1 6.4 (6.0–6.8)	5.87 (5.20–6.62)	6.22 (5.29–7.31)	5.78 (5.11–6.54)	5.72 (4.86–6.73)
PARAGON-HF	205/736 (27.9%) 205/21.0 9.8 (8.5–11.2)	86/325 (26.5%) 86/9.2 9.3 (7.4–11.5)	400/3735 (10.7%) 400/108.4 3.7 (3.3–4.1)	5.49 (4.61–6.53)	5.52 (4.35–7.00)	5.14 (4.30–6.15)	5.21 (4.10–6.64)
ATMOSPHERE	543/1125 (48.3%) 543/34.2 15.9 (14.6–17.3)	283/650 (43.5%) 283/20.0 14.2 (12.6–15.9)	1069/5241 (20.4%) 1069/161.8 6.6 (6.2–7.0)	5.90 (5.30–6.56)	5.25 (4.59–6.00)	5.52 (4.95–6.16)	4.90 (4.28–5.61)
ASTRONAUT	121/404 (30.0%) 121/3.3 36.2 (30.1–43.3)	18/95 (18.9%) 18/0.8 22.0 (13.0–34.7)	153/1116 (13.7%) 153/9.6 15.9 (13.5–18.6)	5.00 (3.89–6.42)	3.07 (1.87–5.03)	5.03 (3.85–6.57)	2.84 (1.72–4.70)
RELAX-AHF-2	197/1060 (18.6%) 197/4.8 41.0 (35.4–47.1)	21/126 (16.7%) 21/0.6 35.8 (22.2–54.7)	537/5359 (10.0%) 537/24.7 21.7 (19.9–23.6)	5.11 (4.29–6.08)	4.75 (3.05–7.38)	4.84 (4.04–5.80)	4.44 (2.81–7.00)
ALTITUDE	139/419 (33.2%) 139/12.8 10.9 (9.1–12.8)	88/257 (34.2%) 88/7.9 11.2 (9.0–13.8)	752/7885 (9.5%) 752/263.0 2.9 (2.7–3.1)	8.18 (6.81–9.83)	8.86 (7.09–11.08)	6.80 (5.61–8.25)	7.32 (5.82–9.19)
NAVIGATOR	60/162 (37.0%) 60/9.1 6.6 (5.0–8.5)	21/59 (35.6%) 21/3.2 6.6 (4.1–10.0)	541/9085 (6.0%) 541/556.8 1.0 (0.9–1.1)	13.11 (10.02–17.16)	14.20 (9.17, 21.99)	6.03 (4.48–8.13)	10.72 (6.85–16.79)

ACEi, angiotensin-converting enzyme inhibitor; AF, atrial fibrillation; ARB, angiotensin receptor blocker; BMI, body mass index; EAR, exposure-adjusted rate; eGFR, estimated glomerular filtration rate; HFH, heart failure hospitalization; HR, hazard ratios; MI, myocardial infarction; NE, not estimable.

^a^Includes patients who died on the day of HFH. Time to death calculated from date of randomization. Unadjusted Cox model: confirmed first rehospitalization (yes/no) as time-dependent variable and treatment as a factor was fitted. Adjusted model: confirmed first rehospitalization (yes/no) as time-dependent variable and treatment, age, sex, region, race, pulse, blood pressure, eGFR, BMI, AF status, MI status, diabetic status, beta blocker, ACEi/ARB, and aldosterone prior use as covariates. Subjects who died on the day of rehospitalization were classified in the ‘No event’ subgroup. *n*/*N*: number of deaths/total number of subjects with rehospitalization; *n*/*T*: number of deaths/total number of 100 patient-years.

### Correlation between log(hazard ratios) based on investigator-reported clinical events and positively adjudicated clinical events

**Figure 1:**
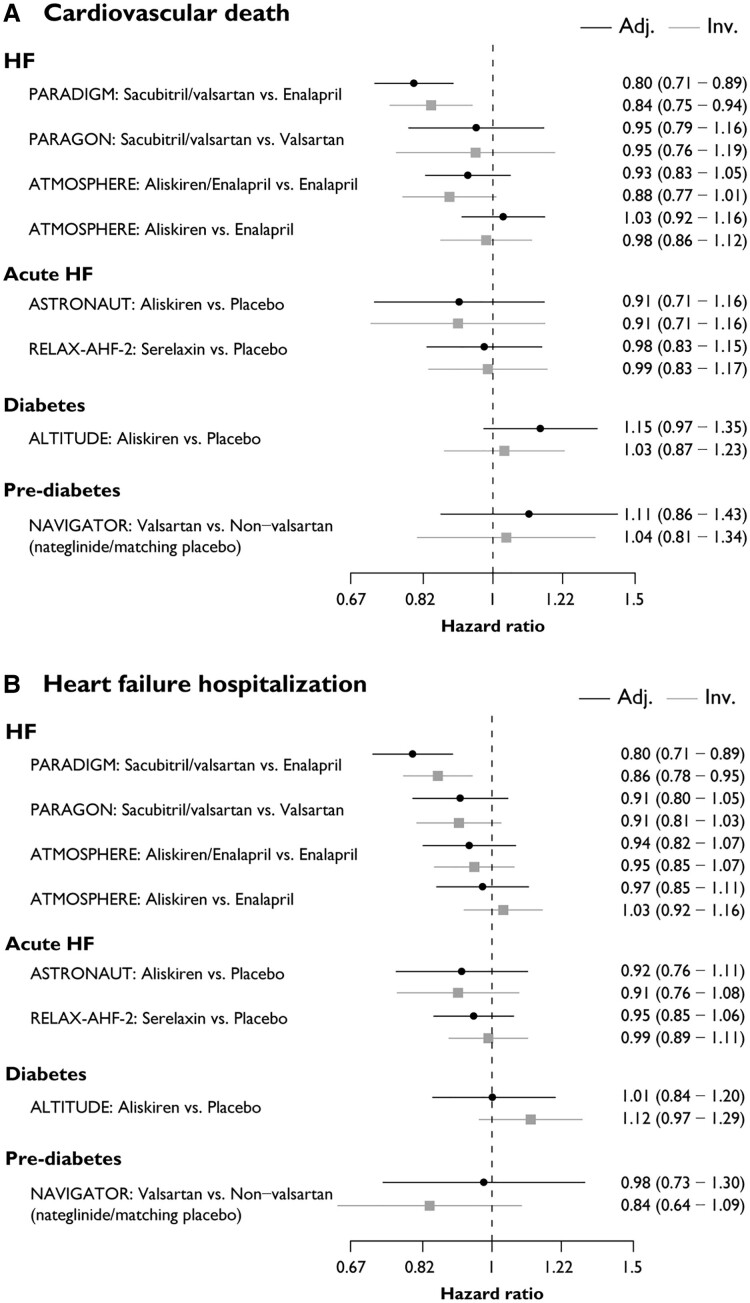

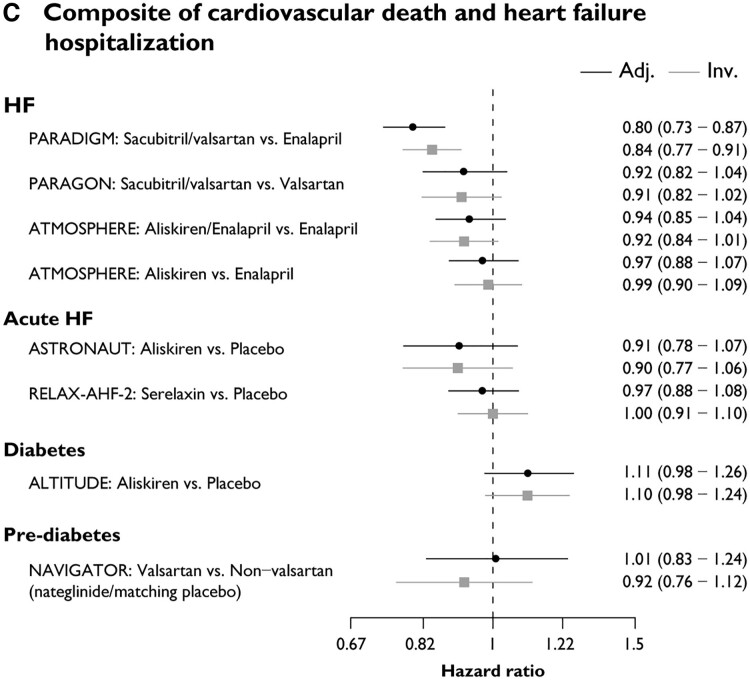
Forest plot of HRs using adjudicated and investigator-reported events for (*A*) CV death, (*B*) HFH, and (*C*) composite of CV death and HFH. Adj., adjudication-confirmed events; CV, cardiovascular; HF, heart failure; Inv., investigator-reported events; vs., versus

Across all studies, estimated log(HR) were generally consistent in magnitude and precision in analyses using adjudicated vs. unadjudicated data for CV death and HFH (*Figure 1*). Treatment by region interaction effects from Cox models showed similar deviation from the overall effect for investigator-reported and adjudicated events (see Supplementary data online, *Figure S1*).

### Correlation between hazard ratios

**Figure 2:**
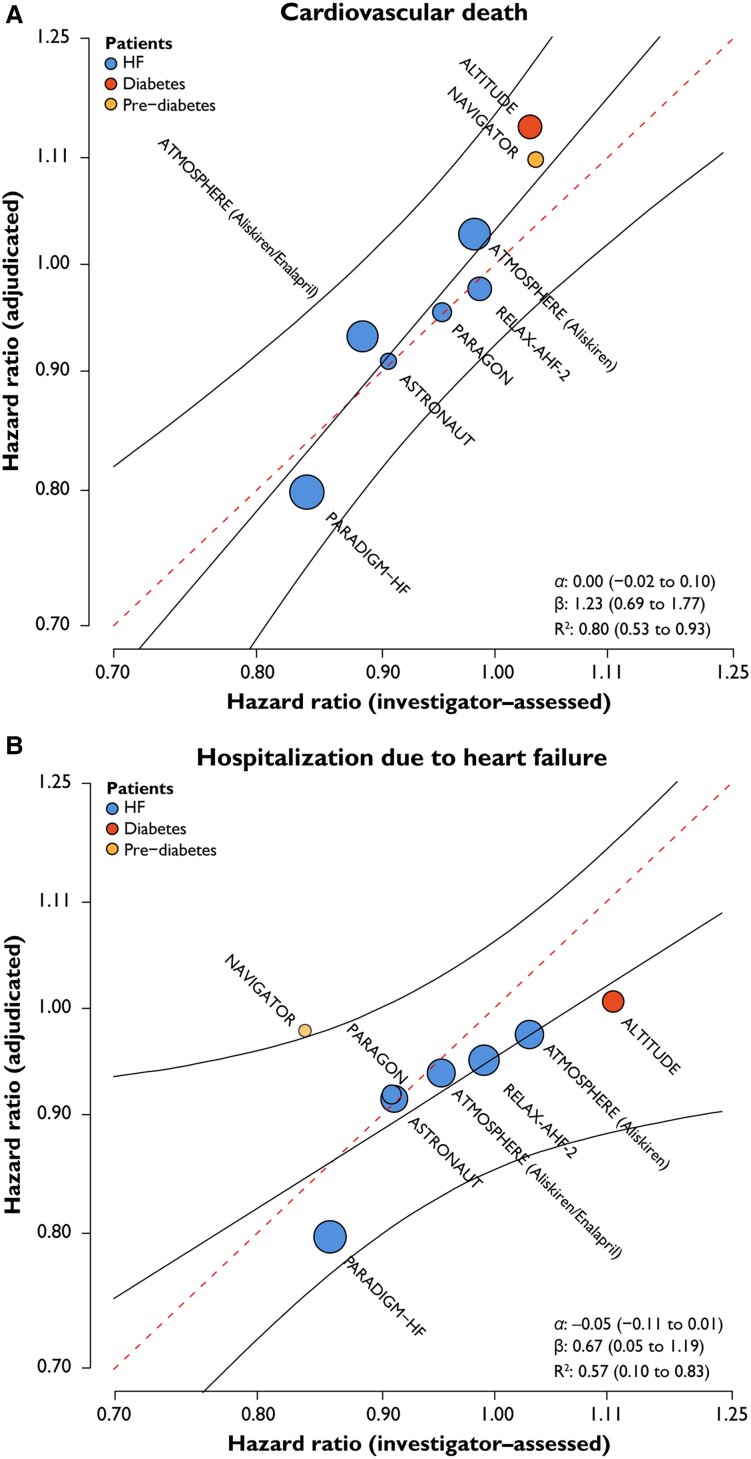

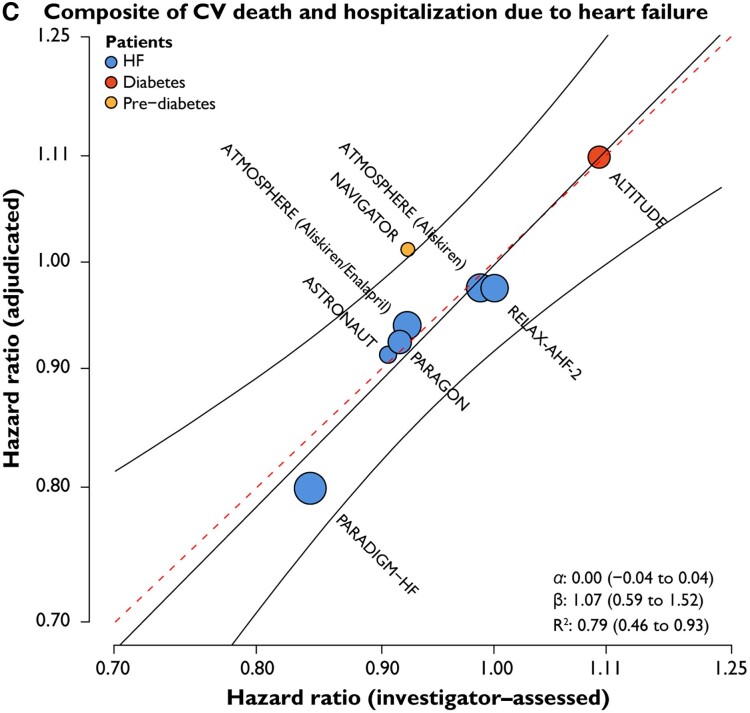
Correlation between HRs using adjudicated and investigator-reported events for (*A*) CV death, (*B*) HFH, and (*C*) composite of CV death and HFH. The dotted line represents the reference line indicating perfect agreement between HR based on adjudicated and investigator-reported endpoints. The line of best fit, with the corresponding 95% prediction interval, is shown in black. The circles reflect each treatment comparison, with the area of the circle being proportional to the amount of information. α: estimated intercept, β: estimated slope. Perfect agreement would occur if α = 0 and β = 1, and CrIs excluding these values would provide evidence against perfect agreement. CV, cardiovascular; HF, heart failure; HFH, heart failure hospitalization; HR, hazard ratios; MI, myocardial infarction; *R*^2^, coefficient of determination

In a meta-regression conducted across studies, log(HR) based on investigator-reported events correlated well with those based on centrally adjudicated events for CV death (*R*^2^ = .80, 95% Crl: 0.53–0.93) and the composite of CV death or HFH (*R*^2^ = .79, 0.46–0.93), but were less correlated for HFH alone (*R*^2^ = .57, 0.10–0.83 ) (*Figure 2*) This was further supported by the estimated intercepts (α) and regression slopes (β), respectively. All CrIs overlapped with perfect agreement, i.e. they included 0 (for α) and 1 (for β; *Figure 2*) simultaneously. The information gain, reported as median (min; max), was −11.2% (−37.7%; 2.7%) for CV death, 23.6% (2.7%; 29.7%) for HFH, and 7.7% (0.8%; 10.9%) for the composite endpoint. Thus, there was slightly higher precision for the estimate of the log(HR) when using adjudicated CV death events, yet the opposite was the case for HFH and the composite endpoint, with substantial gains in precision for HFH when using investigator-reported events.

## Discussion

In this meta-analysis of data from seven large, randomized CV outcome trials, positive adjudication rates varied widely by study and were lower for investigator-reported HF events than for CV death. Investigator-reported HF events were associated with a similar risk of subsequent all-cause mortality whether or not they were positively adjudicated by the CEC. Estimated log(HR) in analyses using investigator-reported vs. centrally adjudicated events were well correlated for CV death and the composite of CV death or HFH, but less so for HFH alone (*Structured Graphical Abstract* ). For trials enrolling patients with HF, centralized adjudication did not substantially change the accuracy of log(HR) with regard to composite HF outcomes, but there was greater variation observed in trials enrolling patients with (pre-)diabetes. Given this variable impact of centralized adjudication on study results across trials, the decision to deploy centralized adjudication likely needs to be individualized, with accounting for the expertise of investigators, the study population being examined, and the process by which potential events are identified for centralized review. The geographical representation may also be considered, since agreement rates for HFH (though not for CV death) were lower in Latin America, yet there seemed to be no impact on the log(HR) (see Supplementary data online, *Table S3* and Supplementary data online, *Figure S1*). Consequently, it may be adequate for certain trials to rely on investigator-reported events only. This is in line with the recommendations made by Khan *et al*.^28^

Recognizing that therapies targeting the CV system may not influence non-CV causes of hospitalization or death, to ensure adequate power, CV outcome trials increasingly rely on composite primary endpoints that emphasize CV death and non-fatal CV events, such as myocardial infarction, HFH, or stroke.^1^ These more specific endpoints are more vulnerable to the subjective judgement of individual investigators and ascertainment may be influenced by local variations in clinical practice. Accordingly, there has been a strong preference for centralized adjudication of CV death and CV events in trials to enhance standardization and limit imprecision introduced by the ‘noise’ of events that might be insensitive to the treatment under study and could dilute important signals of benefit or harm.^1^ Heart failure endpoints seem particularly vulnerable to misclassification due to symptom overlap with comorbid medical conditions and challenges in ascertainment of physical exam signs, as well as challenges in differentiating the primary cause of hospitalization, most importantly in cases where multiple factors may drive admission or when HF complicates admission for another primary cause.^6^

Although this argument is intuitively appealing, there is limited objective evidence to support the premise that adjudicated results are more valid or accurate than unadjudicated results, especially in randomized trials where the ‘noise’ introduced by misclassification of events should be evenly distributed between treatment arms.^9,29^ Rates of concordance between investigator-reported events and CEC-adjudicated events may provide some insight into the value of centralized review, and in our study varied widely by the type of event and the population under study. Although concordance rates for adjudication of CV death were generally >90% in most studies, rates of concordance for HF events were much lower, particularly in studies of (pre-)diabetes and chronic HF, for which as many as 25%–30% of events were negatively adjudicated by the CEC. There are a number of potential reasons for this discordance: in order to capture all events, investigators are commonly encouraged to maintain a low threshold for submitting events for adjudication, and site monitors may trigger additional reporting of potential events based on review of suspicious adverse events. Since this initial sweep is intentionally wide, some degree of editing on further review by the CEC is desirable and should be anticipated; however, the positive adjudication rate is also influenced by the quality of information provided by sites and the specific criteria that are applied to define study endpoints.^1^ For HF events in particular, the commonly utilized criteria defined by the Standardized Clinical Trials Initiative are highly specific, and may be difficult to substantiate in some cases where data are limited.^1^ In the PARAGON-HF trial, for example, nearly 92% of negatively adjudicated cases were ascribed to the lack of adequate source documentation to substantiate the sign, symptom, and treatment criteria needed to certify an HF event.^7^ Our data highlight that these negatively adjudicated events remain prognostically relevant for patients, perhaps because hospitalization for any reason in HF trials is associated with a higher risk of mortality,^1,30^ but also because some of these may be real HF events where data limitations precluded CEC confirmation. Readjudication of data from the PARAGON-HF trial suggested that application of less stringent criteria might permit inclusion of a large proportion of negatively adjudicated HF events.^7^ Collectively, these data raise concerns that the CEC process may inadvertently remove ‘true’ events reported by the investigators, and potentially prolong study duration by amplifying the time needed to accrue the target number of primary endpoints. The event definition and the completeness of source documents thus play an important role in the adjudication process and influence the agreement rates.

Perhaps more important than concordance rates, however, is the degree to which adjudication truly influences the magnitude and accuracy of treatment effect estimates in clinical trials.^2,31^ Our data suggest that for CV death and the composite of CV death or HFH, treatment effect estimates in terms of log(HR) are well correlated in analyses using investigator-reported and centrally adjudicated data, arguing against a strong impact of centralized adjudication on these study results. For the endpoint of HFH alone, however, there was more variability across studies, with less correlation in meta-regression between log(HR) estimated from adjudicated vs. investigator-reported data, particularly for trials of (pre-)diabetes and chronic HF. While in most cases, the observed variation in estimated log(HR) was small, this difference was statistically relevant to the interpretation of overall study results in PARAGON-HF, where a ‘borderline’ result based on adjudicated HF data was nominally positive on analysis of investigator-reported HFHs and on readjudication by experts applying less stringent, probabilistic criteria.^7,32^ By confirming events that have been reported by investigators, CECs promote specificity but may fail to confirm true events that for various reasons, including lack of adequate source documentation, could not be verified, thereby decreasing the sensitivity of the process.^9,32,33^ These data, which are consistent with results from the recent analysis of outcomes from the SHIFT trial^2,34^ and prior meta-analyses of investigator-reported vs. centrally adjudicated events in other trials,^9,35^ suggest that the impact of centralized adjudication on treatment effect estimates in individual trials may vary according to the trial and the specifics of the process for event ascertainment and CEC review. Given the cost and complexity of centralized adjudication, as well as the potential impacts on study duration, these data raise important questions about the need for routine adjudication of HF events in randomized CV outcome trials.^9,32^

However, the analysis should be viewed in the context of important limitations. These data examining CV death and HF outcomes from seven randomized trials supported by a single sponsor may not be generalizable to other endpoints or study designs. Additionally, only one study each in diabetes/pre-diabetes was available, which limits the generalizability of findings for these indications. Centralized adjudication may be more crucial in non-randomized or unblinded trials in which endpoint ascertainment may be more vulnerable to bias,^31^ and for endpoints that are more prone to misclassification (e.g. outpatient worsening of HF, transient ischaemic attack, hospitalization for unstable angina, myocardial infarction). In addition, it remains an inherent challenge of the event adjudication process, that the bedside experience of the treating physician (patient presentation, verbal communication, feedback/observations of other caregivers etc.) can never be fully reflected in the case report forms. Moreover, observed variations in concordance rates and the precision of treatment effect estimates across trials of acute HF, (pre-)diabetes, and chronic HF suggest that the value of centralized review may vary depending on the population under study and the expertise of the investigators (or their study coordinators) who are charged with initial ascertainment of events.^1,2^ The broader the ‘funnel’ of events that is initially cast for potential events, the greater may be the importance of centralized adjudication. These data from studies conducted over a wide timespan also may not accurately reflect the evolution of the CEC process over time, which has been tuned to less stringently apply adjudication criteria and allow room for clinical judgement in cases that meet the ‘spirit’ but not the ‘letter’ of the law.^1^ Finally, these data do not account for other potentially relevant functions of centralized adjudication, including identification of events not reported by investigators (CEC-identified events),^2^ further sub-classification of cause of death or hospitalization, and auxiliary data collection (e.g. the contribution of COVID-19 to CV events) that may influence the subsequent interpretation of study results.

Nonetheless, these data provide further evidence that despite discordance between CEC-adjudicated and investigator-reported events routine use of centralized adjudication for CV death and HFH events does not substantially alter the precision or magnitude of treatment effect estimates in terms of log(HR) in many CV trials. Moving forward, the decision to centrally adjudicate HF events in trials should be individualized and must reflect careful consideration of the benefits of CEC adjudication (consistency, face validity, additional data) and the costs (longer study duration, greater operational complexity) in the context of the expertise of the investigators and the anticipated challenge of endpoint ascertainment in the population under study. These data should also give comfort to CECs that a more liberal or permissive construction of adjudication criteria for HF events that allows room for best clinical judgement and gives the ‘benefit of the doubt’ to investigators in certifying events is unlikely to negatively influence study results.

## Supplementary Material

ehae753_Supplementary_Data

## Declarations

### Data Availability

Novartis is committed to sharing with qualified external researchers, access to patient-level data, and supporting clinical documents from eligible studies. These requests are reviewed and approved by an independent review panel on the basis of scientific merit. All data provided are anonymized to respect the privacy of patients who have participated in the trial in line with applicable laws and regulations. These trial data availability are according to the criteria and process described on www.clinicalstudydatarequest.com.

### Pre-registered Clinical Trial Number

None. Since this is a secondary meta-analysis, it is not registered on a clinical trials registry. The individual trials included in this meta-analysis are registered on a clinical trials registry.
