# Supplementary material for: Centrally adjudicated vs. investigator-reported outcomes in randomized heart failure trials
Source: Eur Heart J. 2024 Nov 9;45(47):5087–99. doi: 10.1093/eurheartj/ehae753 (PMC11646619; doi:10.1093/eurheartj/ehae753)
Supplement: ehae753_Supplementary_Data [file ehae753_supplementary_data.docx]

# **Supplementary data online**

## **PRISMA IPD flow diagram**

**
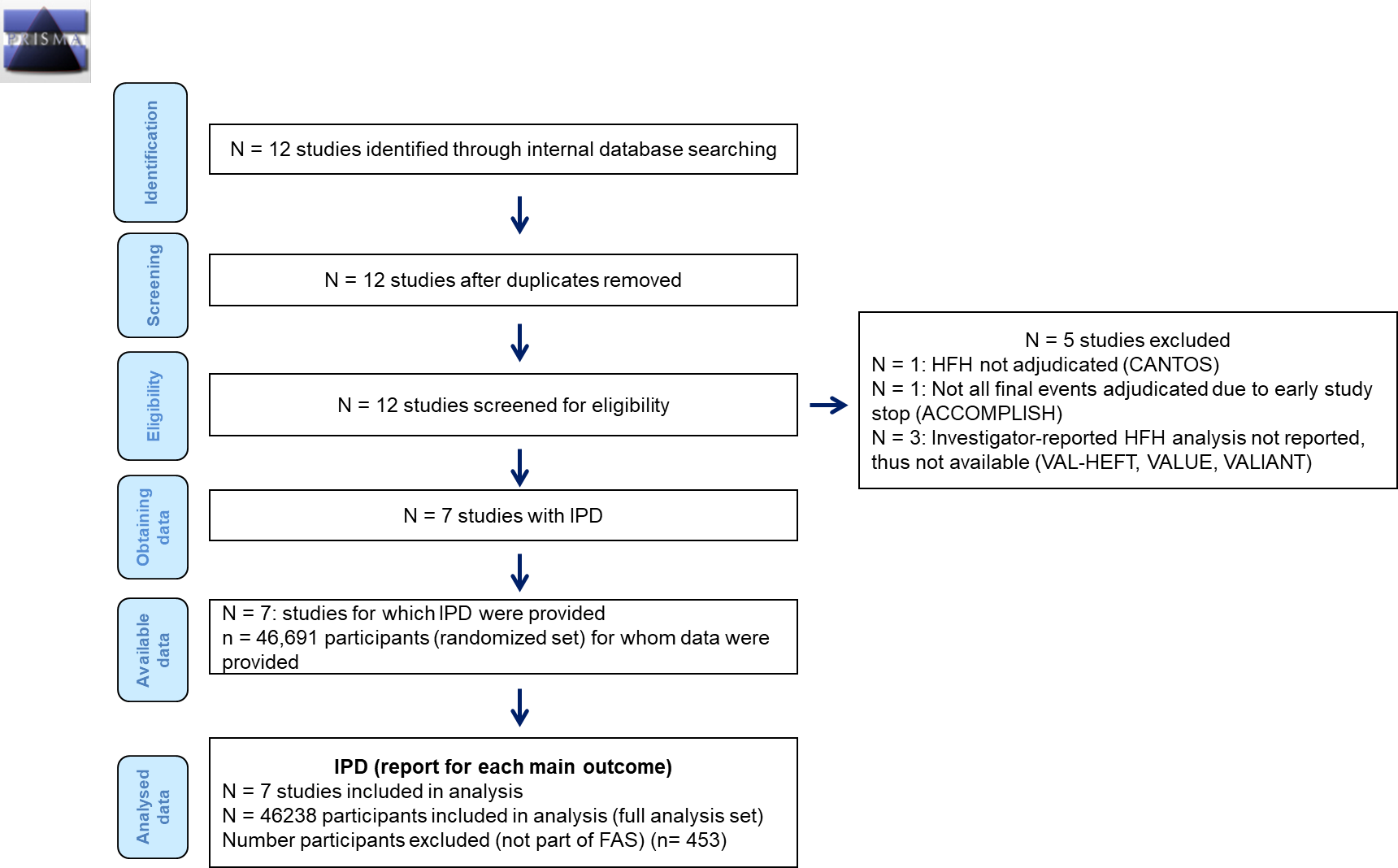
**

## **Statistical analyses: Details on Daniels and Hughes model, bootstrap and** $\boldsymbol{R}^{\mathbf{2}}$

Here are some elementary definitions. The log hazard ratio (HR) from the Cox proportional hazards model is used as the measure of the treatment effect. For the surrogacy assessment, the true endpoint is based on the adjudicated and surrogate endpoint on the investigator-assessed events. Furthermore, the study is denoted by $i$, the observed treatment effect for the true (adjudicated) endpoint by $\hat{\theta}_{i}$, and the observed treatment effect for the surrogate (investigator-reported) endpoint by $\hat{\gamma}_{i}$. The corresponding SEs and correlation are denoted by $\sigma_{i}, \delta_{i}$ and $\rho_{i}$, respectively.

The Daniels and Hughes model is essentially an ‘error in variable’ regression model. For a two-arm study, the model is given by:

$$\binom{\hat{\theta}_{i}}{\hat{\gamma}_{i}}\sim MVN\left( \binom{\theta_{i}}{\gamma_{i}},\left( \begin{matrix} \sigma_{i}^{2} & \rho_{i}\sigma_{i}\delta_{i} \\ \rho_{i}\sigma_{i}\delta_{i} & \delta_{i}^{2} \end{matrix} \right) \right)$$

where the treatment effect for the true endpoint is modelled as a linear function of the treatment effect for the surrogate endpoint:

$$\theta_{i}|\gamma_{i}\sim N(\alpha+\beta\gamma_{i}, \tau^{2})$$

This model can easily be extended to multi-arm studies; in the current case, an extension up to three arms (two treatment effects) is required, i.e.:

$$\left( \begin{matrix} \begin{matrix} \hat{\theta}_{i1} \\ \hat{\theta}_{i2} \end{matrix} \\ \begin{matrix} \hat{\gamma}_{i1} \\ \hat{\gamma}_{i2} \end{matrix} \end{matrix} \right)\sim MVN\left( \left( \begin{matrix} \begin{matrix} \theta_{i1} \\ \theta_{i2} \end{matrix} \\ \begin{matrix} \gamma_{i1} \\ \gamma_{i2} \end{matrix} \end{matrix} \right),\left( \begin{matrix} \begin{matrix} \sigma_{i1}^{2} & \rho_{i\theta_{12}}\sigma_{i1}\sigma_{i2} \\ \rho_{i\theta_{12}}\sigma_{i1}\sigma_{i2} & \sigma_{i2}^{2} \end{matrix} & \begin{matrix} \rho_{i11}\sigma_{i1}\delta_{i1} & \rho_{i11}\sigma_{i1}\delta_{i2} \\ \rho_{i21}\sigma_{i2}\delta_{i1} & \rho_{i22}\sigma_{i2}\delta_{i2} \end{matrix} \\ \begin{matrix} \rho_{i11}\sigma_{i1}\delta_{i1} & \rho_{i21}\sigma_{i2}\delta_{i1} \\ \rho_{i11}\sigma_{i1}\delta_{i2} & \rho_{i22}\sigma_{i2}\delta_{i2} \end{matrix} & \begin{matrix} \delta_{i1}^{2} & \rho_{i\gamma_{12}}\delta_{i1}\delta_{i2} \\ \rho_{i\gamma_{12}}\delta_{i1}\delta_{i2} & \delta_{i2}^{2} \end{matrix} \end{matrix} \right) \right)$$

Here, for study $i$,

- $\rho_{i\theta_{..}}$ denotes the correlation between treatment effects for the true endpoint
- $\rho_{i\gamma_{..}}$ denotes the correlation between treatment effects for the surrogate endpoint
- $\rho_{i}..$ denotes the correlation between treatment effects across the true and the surrogate endpoint

The relationship between the true and the surrogate endpoint is then

$$\theta_{ik}|\gamma_{ik}\sim N(\alpha+\beta\gamma_{ik}, \tau^{2})$$

As is typically done, it was assumed that the SEs ${\sigma_{ik}, \delta}_{ik}$ and the correlations $\rho_{i\theta.,}\rho_{i\gamma.}, \rho_{i..}$ are known. While the SEs are directly obtained from the Cox models, the correlations are obtained through bootstrapping, using 1000 samples with replacement. Therefore, it was only required to specify the following prior distributions (all of them weakly informative):

$$\begin{matrix} \alpha\sim N\left( 0, 2^{2} \right) \\ \beta\sim N\left( 0, 2^{2} \right) \\ \begin{matrix} \gamma_{ik}\sim N\left( {0,2}^{2} \right) \\ \tau\sim HN(0.5) \end{matrix} \end{matrix}$$

Here, $HN(s)$ is the half-normal distribution with scale $s$, i.e. if $x \sim N\left( 0,s^{2} \right)$ then $\left| x \right|\sim HN(s)$. The motivation for using these priors for HRs follows similar arguments as outlined for log odds ratios in Friede et al^5^.

The model was implemented in JAGS, for which the code is given as an R function below. The data structure for this model is essentially a ‘wide’ structure, with the following data being required:

- nstudies: number of studies (scalar)
- nparms: vector of length nstudies, each entry determining the number of parameters of the study (i.e. twice the number of treatment comparisons)
- m: matrix of dimension nstudies x max(nparms[ ]), observed treatment effects for the true and surrogate endpoint, respectively. E.g. in our case, if the first is a two-arm study, it will be m[1,1:6] = c(..., ..., NA, NA, NA, NA).
- prec: array of dimensions nstudies x max(nparms[ ]) x max(nparms[ ]), observed **precision** (i.e. inverse variance) matrices. E.g. in the current case, if the first is a two-arm study, prec[1,1:6,1:6] will be NA except for prec[1,1:2,1:2] which will be the precision matrix for the first study
- ntrtc: vector of length nstudies, each entry determining the number of treatment comparisons of the study
- m.alpha, prec.alpha, m.beta, prec.beta, m.gamma, prec.gamma: each a scalar, mean and precision for prior distributions of alpha, beta, and gamma[i,k]
- inv.squared.scale: the precision (inverse of squared scale) of the half-normal prior distribution for tau

mod <- function(){

for(i in 1:nstudies){

m[i,1:nparms[i]] ~ dmnorm(mn[i,1:nparms[i]], prec[i,1:nparms[i],1:nparms[i]])

mn[i,1:ntrtc[i]] <- theta[i,1:ntrtc[i]]

mn[i,(ntrtc[i] + 1):nparms[i]] <- gamma[i,1:ntrtc[i]]

for(k in 1:ntrtc[i]){

gamma[i,k] ~ dnorm(gamma.m, gamma.prec)

theta[i,k] ~ dnorm(theta.m[i,k], prec.tau)

theta.m[i,k] <- alpha + beta*gamma[i,k]

}

}

alpha ~ dnorm(m.alpha, prec.alpha)

beta ~ dnorm(m.beta, prec.beta)

prec.tau <- pow(tau, -2)

tau ~ dnorm(0, inv.squared.scale)%_%T(0,)

pred ~ dnorm(0, prec.tau)

}

For the Bayesian $R^{2}$, the proposal by Gelman et al^7^was followed which is defined it as:

$$R^{2}=\frac{Explained variance}{Explained variance+Residual variance}$$

In fact, the Bayesian $R^{2}$ will have a posterior distribution as well, since both the explained and the residual variance are a function of the model parameters. Therefore, for each MCMC draw $s$, the above can be written as:

$$R_{s}^{2}=\frac{Explained variance_{s}}{Explained variance_{s}+Residual variance_{s}}=\frac{V_{1}^{N}y_{n}^{pred s}}{V_{1}^{N}y_{n}^{pred s}+ var_{res}^{s}}$$

To reflect the fact that the weight (amount of information) differs between the $y_{n}$, because these reflect treatment comparisons, inverse variance weights were used based on the variance of the adjudicated treatment effect and the random effect variance. The weighted variances can then be calculated. Finally, it was noted that the above $R^{2}$ is still an approximation, since the uncertainty of the explanatory variable is ignored (treatment effect based on investigator-reported endpoint).

## **Table S1: CV death definition as per CEC**

| **Study name** | **Study design** |
| --- | --- |
| HFrEF and HFpEF | |
| PARADIGM-HF | Cardiovascular death includes death classified in any of the following categories:  **1. Fatal Myocardial Infarction (MI):**  Fatal MI may be adjudicated in any of the following three scenarios:  a. Death occurring within 14 days after a documented MI, in which there is no conclusive evidence of any other cause of death. Subjects who are being treated for a MI and who die as a result of complications of the MI (eg, sudden death, pump failure, or cardiogenic shock) will be classified as having had a MI-related death.  b. Autopsy evidence of a recent infarct with no conclusive evidence of any other cause of death  c. An abrupt death that has characteristics suggestive of an acute infarct but do not meet the strict definition of a MI. Suggestive characteristics are:  ***presentation with acute ischemic symptoms***  ***AND one of the following:***  o ***ECG changes indicative of an acute injury***  o ***abnormal cardiac biomarkers***  o ***other evidence (eg, echocardiography, ventriculography, or scintigraphy) of new ventricular wall motion abnormality***  **2. Pump Failure:**  Death occurring in the context of clinically worsening symptoms and/or signs of heart failure (HF) without evidence of another cause of death.  Death occurring as a complication of the implantation of a ventricular assist device, cardiac transplant, or other surgery primarily for refractory HF.  Death occurring after referral to hospice specifically for progressive HF.  *Note:* If worsening HF is secondary to MI, then MI should be listed as the primary cause of death if the subject suffered an MI within 14 days of death (as above).  **3. Sudden Death:**  Death occurring unexpectedly in an otherwise stable subject. Further subclassification of sudden death will be as follows:   1. death witnessed or subject last seen alive <1 hour previously ***or*** (2) subject last seen alive >1 hr and <24 hrs previously   **4. Presumed Sudden Death**  Death occurring unexpectedly in an otherwise stable subject last seen alive ≥ 24 hours previously, with circumstances suggestive of sudden death***.***  **5. Presumed Cardiovascular Death:**  Death likely due to a cardiovascular cause in which the available clinical data is insufficient to support a more specific cause of death.  **6. Fatal Stroke**:  Death occurring as a result of a documented stroke. Where possible, the stroke will be further classified as ischemic, ischemic with hemorrhagic conversion, primary intracranial haemorrhage, or unknown.    **7. Fatal Pulmonary Embolism:**  Death occurring as a direct result of a documented pulmonary embolism.  **8. Procedure-Related Death:**  Death occurring during a cardiovascular procedure or as a result of complications related to a cardiovascular procedure (e.g. percutaneous coronary intervention), usually within 14 days.  **9. Other Cardiovascular Death:**  Death resulting from a specifically documented cardiovascular cause other than those listed above. |
| ATMOSPHERE | **Cardiovascular Death**  Cardiovascular death is defined as follows:  **1. Fatal Myocardial Infarction (MI):**  Fatal MI may be adjudicated in any of the following three scenarios:  a. Death occurring within 14 days after a documented MI, in which there is no conclusive evidence of any other cause of death. Subjects who are being treated for a MI and who die as a result of complications of the MI (eg, sudden death, pump failure, or cardiogenic shock) will be classified as having had a MI-related death.  b. Autopsy evidence of a recent infarct with no conclusive evidence of any other cause of death  c. An abrupt death that has characteristics suggestive of an acute infarct but do not meet the strict definition of a MI. Suggestive characteristics are:  ♦ ***presentation with acute ischemic symptoms***  ♦ ***AND one of the following:***  o ***ECG changes indicative of an acute injury***  o ***abnormal cardiac biomarkers***  o ***other evidence (eg, echocardiography, ventriculography, or scintigraphy) of new ventricular wall motion abnormality***  **2. Pump Failure:**  Death occurring in the context of clinically worsening symptoms and/or signs of HF without evidence of another cause of death.  Death occurring after the implantation of a ventricular assist device or after surgery primarily for refractory HF.  Death occurring after referral to hospice specifically for progressive HF.  *Note:* If worsening HF is secondary to MI, then MI should be listed as the primary cause of death if the subject suffered an MI within 14 days of death (as above).  **3. Sudden Death:**  Death occurring unexpectedly in an otherwise stable subject. Further subclassification of sudden death will be as follows:  (1) death witnessed or subject last seen alive <1 hour previously ***or***  (2) subject last seen alive ≥1 hr and <24 hrs previously  **4. Presumed Sudden Death**  Death occurring unexpectedly in an otherwise stable subject last seen alive ≥24 hours previously, with circumstances suggestive of sudden death***.***  **5. Presumed Cardiovascular Death:**  Death likely due to a cardiovascular cause in which the available clinical data is insufficient to support a more specific cause of death.  **6. Fatal Stroke**:  Death occurring as a result of a documented stroke. Where possible, the stroke will be further classified as ischemic, ischemic with hemorrhagic conversion, primary intracranial haemorrhage, or unknown.  **7. Fatal Pulmonary Embolism:**  Death occurring as a direct result of a documented pulmonary embolism.  **8. Procedure-Related Death:**  Death occurring during a cardiovascular procedure or as a result of procedure-related complications, usually within 14 days.  **9. Other Cardiovascular Death:**  Death resulting from a specifically documented cardiovascular cause other than those listed above. |
| PARAGON-HF | **Cardiovascular Death**  Cardiovascular death includes death classified in any of the following categories:  **1. Fatal Myocardial Infarction (MI):**  Fatal MI may be adjudicated in any of the following three scenarios:  a. Death occurring within 14 days after a documented MI, in which there is no conclusive evidence of any other cause of death. Subjects who are being treated for a MI and who die as a result of complications of the MI (eg, sudden death, pump failure, or cardiogenic shock) will be classified as having had a MI-related death.  b. Autopsy evidence of a recent infarct with no conclusive evidence of any other cause of death  c. An abrupt death that has characteristics suggestive of an acute infarct but do not meet the strict definition of a MI. Suggestive characteristics are:  ***presentation with acute ischemic symptoms***  ***AND one of the following:***  o ***ECG changes indicative of an acute injury***  o ***abnormal cardiac biomarkers***  o ***other evidence (eg, echocardiography, ventriculography, or scintigraphy) of new ventricular wall motion abnormality***  **2. Heart Failure:**  Death occurring in the context of clinically worsening symptoms and/or signs of heart failure (HF) without evidence of another cause of death.  Death occurring as a complication of the implantation of a ventricular assist device, cardiac transplant, or other surgery primarily for refractory HF.  Death occurring after referral to hospice specifically for progressive HF.  *Note:* If worsening HF is secondary to MI, then MI should be listed as the primary cause of death if the subject suffered an MI within 14 days of death (as above).  **3. Sudden Death:**  Death occurring unexpectedly in an otherwise stable subject. Further subclassification of sudden death will be as follows:  a. death witnessed or subject last seen alive <1 hour previously ***or***  b. subject last seen alive ≥1 hr and <24 hrs previously  **4. Presumed Sudden Death:**  Death occurring unexpectedly in an otherwise stable subject last seen alive ≥24 hours previously, with circumstances suggestive of sudden death***.***  **5. Presumed Cardiovascular Death:**  Death likely due to a cardiovascular cause in which the available clinical data is insufficient to support a more specific cause of death.  **6. Fatal Stroke**:  Death occurring as a result of a documented stroke. Where possible, the stroke will be further classified as ischemic, ischemic with hemorrhagic conversion, primary intracranial haemorrhage, or unknown.    **7. Fatal Pulmonary Embolism:**  Death occurring as a direct result of a documented pulmonary embolism.  **8. Cardiovascular Procedure-Related Death:**  Death occurring during a cardiovascular procedure or as a result of complications related to a cardiovascular procedure (e.g. percutaneous coronary intervention), usually within 14 days. The CEC will subcategorize these deaths as related to percutaneous coronary intervention (**PCI-related**), coronary artery bypass-grafting (**CABG-related**), valvular procedures (**valvular)**, or other cardiovascular procedures (**other**).  **9. Other Cardiovascular Death:**  Death resulting from a specifically documented cardiovascular cause other than those listed above. |
| **Acute HF (HFrEF and HFpEF)** | |
| ASTRONAUT | **Cardiovascular Death**  Cardiovascular death is defined as follows:  **1. Fatal Myocardial Infarction (MI):**  Fatal MI may be adjudicated in any of the following three scenarios:  a. Death occurring within 14 days after a documented MI, in which there is no conclusive evidence of any other cause of death. Subjects who are being treated for a MI and who die as a result of complications of the MI (eg, sudden death, pump failure, or cardiogenic shock) will be classified as having had a MI-related death.  b. Autopsy evidence of a recent infarct with no conclusive evidence of any other cause of death  c. An abrupt death that has characteristics suggestive of an acute infarct but do not meet the strict definition of a MI. Suggestive characteristics are:  ♦ ***presentation with acute ischemic symptoms***  ♦ ***AND one of the following:***  o ***ECG changes indicative of an acute injury***  o ***abnormal cardiac biomarkers***  o ***other evidence (eg, echocardiography, ventriculography, or scintigraphy) of new ventricular wall motion abnormality***  **2. Pump Failure:**  Death occurring in the context of clinically worsening symptoms and/or signs of HF without evidence of another cause of death  Death occurring after the implantation of a ventricular assist device or after surgery primarily for refractory HF  Death occurring after referral to hospice specifically for progressive heart failure  *Note:* If worsening HF is secondary to MI, then MI should be listed as the primary cause of death if the subject suffered an MI within 14 days of death (as above).  **3. Sudden Death:**  Death occurring unexpectedly in an otherwise stable subject. Further subclassification of sudden death will be as follows:  (1) death witnessed or subject last seen alive <1 hour previously ***or***  (2) subject last seen alive ≥1 hr and <24 hrs previously  **4. Presumed Sudden Death:**  Death occurring unexpectedly in an otherwise stable subject last seen alive ≥ 24 hours previously, with circumstances suggestive of sudden death***.***  **5. Presumed Cardiovascular Death:**  Death likely due to a cardiovascular cause in which the available clinical data is insufficient to support a more specific cause of death.  **6. Fatal Stroke**:  Death occurring as a result of a documented stroke. Where possible, the stroke will be further classified as ischemic, ischemic with hemorrhagic conversion, primary intracranial haemorrhage, or unknown.  **7. Fatal Pulmonary Embolism:**  Death occurring as a direct result of a documented pulmonary embolism.  **8. Procedure-Related Death:**  Death occurring during a cardiovascular procedure or as a result of procedure-related complications, usually within 14 days.  **9. Other Cardiovascular Death:**  Death resulting from a specifically documented cardiovascular cause other than those listed above. |
| RELAX-AHF-2 | **Definitions used to classify cardiovascular deaths**  **Death due to Heart Failure / Cardiogenic Shock (Pump failure)*:***  Death occurring in the context of clinically worsening symptoms and/or signs of heart failure without evidence of another cause of death. New or worsening signs and/or symptoms of congestive heart failure (CHF) may include any of the following:   - New or increasing symptoms and/or signs of heart failure requiring the initiation of, or an increase in, treatment directed at heart failure or occurring in a patient already receiving maximal therapy for heart failure - Heart failure symptoms or signs requiring continuous intravenous therapy or oxygen administration - Confinement to bed predominantly due to heart failure symptoms - Pulmonary oedema sufficient to cause tachypnoea and distress not occurring in the context of an acute myocardial infarction or as the consequence of a primary arrhythmic event - Cardiogenic shock, manifest as clinical signs and symptoms of hypoperfusion felt to be secondary to cardiac dysfunction, and not occurring in the context of an acute myocardial infarction or as the consequence of a primary arrhythmic event   Patients who are hospitalized and are being actively treated for heart failure and who have a sudden death as the terminal event will be classified as having a heart failure related death.  **Sudden Cardiac Death:**  Death that occurs unexpectedly in a previously stable patient will be adjudicated as witnessed or unwitnessed sudden cardiac deaths:  *Witnessed sudden cardiac death:*   - Witnessed and instantaneous without new or worsening symptoms - Witnessed within 60 minutes of the onset of new or worsening cardiac symptoms - Witnessed and attributed to an identified arrhythmia (e.g., captured on an electrocardiographic (ECG) recording or witnessed on a monitor by either a medic or paramedic) - Subjects unsuccessfully resuscitated from cardiac arrest or successfully resuscitated from cardiac arrest but who die without identification of a non-cardiac aetiology   Note that if a witnessed sudden cardiac death occurs as a complication of another primary cardiac process, eg, cardiogenic shock or acute myocardial infarction, the primary process should be adjudicated as the cause of death.  *Unwitnessed sudden cardiac death:*  An unwitnessed death is one that occurs in a patient who when last seen alive within an observation period of 72 hours:   - Did not manifest another life-threatening non-cardiac disease (e.g., infectious, metabolic disorders); and/or - Did not reveal a cause other than cardiovascular (e.g., trauma) at the scene of death; and/or - Death was ruled cardiovascular in cause on an autopsy report or death certificate, and occurred in the absence of pre-existing circulatory failure or other modes of death.   If an unwitnessed sudden cardiac death occurs, information regarding the patient’s clinical status within the week preceding death should be reviewed for potential insight into the primary cause of death.  **Death due to Acute Coronary Syndrome / Acute Myocardial Infarction:**  Death occurring up to 14 days after a documented acute myocardial infarction [verified either by the diagnostic criteria below for acute myocardial infarction or by autopsy findings showing recent myocardial infarction or recent coronary thrombus] and where there is no conclusive evidence of another cause of death.  Acute coronary syndrome / acute myocardial infarction: At least one of the following biochemical indicators of myocardial necrosis must be present:   - CKMB greater than 2 x the upper limit of the normal (ULN) or - Troponin I or T greater than 2 x ULN   AND at least one of the two following criteria:   - Typical clinical presentation consistent with myocardial infarction defined as typical cardiac ischemic type pain/discomfort or dyspnoea felt to be due to ischemia   OR…   - Typical ECG changes consisting of any of the following: - new abnormal Q waves (or new R waves in lead V1-V2) in at least two consecutive leads - evolving, ischemic ST segment or T wave changes in at least two consecutive leads. - new left bundle branch block.   If death occurs before biochemical confirmation of myocardial necrosis can be obtained, adjudication should be based on clinical presentation and ECG evidence.  Death due to an acute myocardial infarction that occurs as a direct consequence of a cardiovascular investigation/procedure/operation will be classified as death due to cardiac procedure.  **Death due to Cerebrovascular Event:**   - Ischemic stroke - Hemorrhagic stroke or hemorrhagic change - Stroke, unknown mechanism   Death occurring up to 30 days after a suspected or confirmed stroke based on clinical signs and symptoms as well as neuroimaging and/or autopsy, and where there is no conclusive evidence of another cause of death. This category will include death occurring up to 30 days after a stroke that is either due to the stroke or caused by a complication of the stroke***.***  **Death due to Other Cardiovascular Causes:**   - Peripheral vascular disease - Systemic embolus - Pulmonary embolus - Cardiac procedure complication - Vascular procedure complication - Other cardiovascular death, specify   Death must be due to a documented cardiovascular cause not included in the above categories (e.g. pulmonary embolism, other vascular death or as a complication of a cardiovascular intervention).  **Presumed Cardiovascular Death:**  All deaths not attributed to the categories of cardiovascular death and not attributed to a non-cardiovascular cause, are presumed cardiovascular deaths and as such are part of the cardiovascular mortality endpoint. |
| **Diabetes and pre-diabetes** | |
| ALTITUDE | **Cardiovascular Death**  Cardiovascular death is defined as follows:  **1. Fatal Myocardial Infarction (MI):**  Fatal MI may be adjudicated in any of the following three scenarios:  a. Death occurring within 14 days after a documented MI, in which there is no conclusive evidence of any other cause of death. Subjects who are being treated for a MI and who die as a result of complications of the MI (egg, sudden death, pump failure, or cardiogenic shock) will be classified as having had a MI related death.  b. Autopsy evidence of a recent infarct with no conclusive evidence of any other cause of death  c. An abrupt death that has characteristics suggestive of an acute infarct but do not meet the strict definition of a MI. Suggestive characteristics are:   - ***presentation with acute ischemic symptoms*** - ***AND one of the following:***   o ***ECG changes indicative of an acute injury***  o ***abnormal cardiac biomarkers***  o ***other evidence (e.g., echocardiography, ventriculography, or scintigraphy) of new ventricular wall motion abnormality***  **2. Pump Failure:**  Death occurring in the context of clinically worsening symptoms and/or signs of HF without evidence of another cause of death  Death occurring after the implantation of a ventricular assist device or after surgery primarily for refractory HF  *Note:* If worsening HF is secondary to MI, then MI should be listed as the primary cause of death if the subject suffered an MI within 14 days of death (as above).  **3. Sudden Death:**  Death occurring unexpectedly in an otherwise stable subject. Further subclassification of sudden death will be as follows:  (1) death witnessed or subject last seen alive <1 hour previously ***or***  (2) subject last seen alive ≥1 hr and <24 hrs previously  **4. Presumed Sudden Death:**  Death occurring unexpectedly in an otherwise stable subject last seen alive ≥ 24 hours previously, with circumstances suggestive of sudden death***.***  **5. Presumed Cardiovascular Death:**  Death likely due to a cardiovascular cause in which the available clinical data is insufficient to support a more specific cause of death.  **6. Fatal Stroke**:  Death occurring as a result of a documented stroke. Where possible, the stroke will be further classified as ischemic, ischemic with hemorrhagic conversion, primary intracranial haemorrhage, or unknown.  **7. Fatal Pulmonary Embolism:**  Death occurring as a direct result of a documented pulmonary embolism.  **8. Procedure-Related Death:**  Death occurring during a cardiovascular procedure (e.g., a coronary artery bypass graft procedure or a percutaneous coronary angioplasty), or as a result of procedure-related complications within 14 days).  **9. Other Cardiovascular Death:**  Death resulting from a documented cardiovascular cause other than those listed above. |
| NAVIGATOR | **Cardiovascular death**  This category will include the following:  *1.* ***1. Sudden Cardiac Death:*** Death that occurs instantaneously or within 60 minutes of onset of symptoms **and the cause of the death is unknown**. Unobserved death within 60 minutes of last contact will be classified as sudden death. Sudden death may occur in the hospital.  This category will also include post-resuscitation death, defined as follows: Patients in whom a cardiac and/or respiratory arrest occurs within 60 minutes of the onset of cardiac or suspected cardiac symptoms but a) are resuscitated and b) do not regain normal vital functions and c) die more than 60 minutes from the onset of symptoms leading to the arrest  *2****. 2. Myocardial Infarction Death:***  ***a) Definite* -** Death which occurs during the hospitalization for the MI and is related to a cardiac complication (e.g. CHF, arrhythmia, shock) of the acute event. MI is documented by clinical, electrocardiographic and enzyme criteria or angiographic or pathological findings. If patient has a documented MI then dies "suddenly" while making an otherwise normal recovery the death will be classified in this category.  ***b) Probable* -** As above but MI is documented by two of three criteria (ECG, Enzyme, Clinical Setting); or patient presentation in typical clinical setting with chest pain or other findings suggestive of Acute MI in the absence of diagnostic enzyme or ECG changes; or the attending physician states that the patient died from MI but does not provide documentation.  ***C3. Congestive Heart Failure:*** Death from intractable congestive heart failure (Class III or IV) not associated with an acute event.  *4* ***4. Stroke:*** Death in which the primary cause is stroke.  ***5. 5. Other Cardiovascular Cause:*** Death in which there is evidence of a primary cardiovascular aetiology, and does not clearly meet the criteria for the categories outlined above. This category also includes: arrhythmogenic death, cardiac rupture, and vascular death (arterial embolism, pulmonary embolism, spontaneous aortic dissection/rupture, and bleeding).  *6****. 6. Presumed CV death:*** death occurring when the patient was last seen >60 minutes before death and presumed to be cardiovascular.  ***7. Cardiovascular procedure related death:*** death during or within 24 hours following a surgical or percutaneous cardiovascular procedure (e.g. PCI, CABG, etc.) and considered related to the process |

## **Table S2: HFH definition as per CEC**

| **Study name** | **Study design** |
| --- | --- |
| **HFrEF and HFpEF** | |
| PARADIGM-HF | **Hospitalization for Heart Failure (HF)**  Presentation to an acute care facility requiring an overnight hospitalization (change in calendar day) with an exacerbation of heart failure requiring treatment meeting the following criteria:  **1. Symptoms and signs of heart failure:**  One or more of the following symptoms consistent with heart failure:  a. Worsening dyspnoea  b. Worsening orthopnoea  c. Paroxysmal nocturnal dyspnoea  d. Increasing fatigue/ worsening exercise tolerance  e. Worsening oedema/anasarca  **AND**  Two or more of the following signs consistent with heart failure:  a. Rapid weight gain  b. Pulmonary oedema or rales  c. Elevated jugular venous pressure  d. Radiologic signs of heart failure  e. Peripheral oedema  f. Increasing abdominal distension or ascites  g. S3 gallop  h. Hepatojugular reflux  i. Elevated brain natriuretic peptide (BNP) or N-terminal pro-BNP (>most recent baseline)  **AND**  **2. Treatment**  Treatment with intravenous diuretics, intravenous vasodilators, intravenous inotropes, mechanical fluid removal (e.g., ultrafiltration or dialysis), or insertion of an intra-aortic balloon pump for hemodynamic compromise.  Initiation of standing oral diuretics or intensification (doubling) of the maintenance diuretic dose will also qualify as treatment. |
| ATMOSPHERE | **Hospitalization for Heart Failure (HF)**  Presentation to an acute care facility requiring an overnight hospitalization (change in calendar day) with an exacerbation of heart failure requiring treatment meeting the following criteria:  **1. Symptoms and signs of heart failure:**  One or more of the following symptoms consistent with heart failure:  a. Worsening dyspnoea  b. Worsening orthopnoea  c. Paroxysmal nocturnal dyspnoea  d. Increasing fatigue/ worsening exercise tolerance  **AND**  Two or more of the following signs consistent with heart failure:  a. Rapid weight gain  b. Pulmonary oedema or rales  c. Elevated jugular venous pressure  d. Radiologic signs of heart failure  e. Peripheral oedema  f. Increasing abdominal distension or ascites  g. S3 gallop h. Hepatojugular reflux  i. Elevated brain natriuretic peptide (BNP) or N-terminal pro-BNP (>most recent baseline)  **AND**  **2. Treatment**  Treatment with intravenous diuretics, intravenous vasodilators, intravenous inotropes, mechanical fluid removal (e.g., ultrafiltration or dialysis), or insertion of an intra-aortic balloon pump for hemodynamic compromise. Initiation of standing oral diuretics or intensification (doubling) of the maintenance diuretic dose will also qualify as treatment. |
| PARAGON-HF | **Hospitalization for Heart Failure (HF)**  Presentation to an acute care facility requiring an overnight hospitalization (change in calendar day) with an exacerbation of heart failure requiring treatment meeting the following criteria:  **1. Symptoms and signs of heart failure:**  One or more of the following symptoms consistent with heart failure:  a. Increasing dyspnoea  b. Worsening orthopnoea  c. Paroxysmal nocturnal dyspnoea  d. Increasing fatigue/ decreasing exercise tolerance  e. Worsening oedema/anasarca  **AND**  Two or more of the following signs consistent with heart failure:  a. Rapid weight gain  b. Pulmonary oedema or rales  c. Elevated jugular venous pressure  d. Radiologic signs of heart failure  e. Peripheral oedema  f. Increasing abdominal distension or ascites  g. S3 gallop  h. Hepatojugular reflux  i. Elevated brain natriuretic peptide (BNP) or N-terminal pro-BNP (>most recent baseline)  j. Congestive hepatomegaly (i.e. not related to intrinsic liver disease)  k. Invasive/Non-invasive tests showing cardiac filling pressures or low cardiac output  **AND**  **2. Treatment**  Treatment with intravenous diuretics, intravenous vasodilators, intravenous inotropes, mechanical fluid removal (e.g., ultrafiltration or dialysis), or insertion of an intra-aortic balloon pump for hemodynamic compromise. Initiation of standing oral diuretics or intensification (doubling) of the maintenance diuretic dose will also qualify as treatment. |
| **Acute HF (HFrEF and HFpEF)** | |
| ASTRONAUT | **Hospitalization for Heart Failure (HF)**  Presentation to an acute care facility requiring an overnight hospitalization (change in calendar day) with an exacerbation of heart failure requiring treatment meeting the following criteria:  **1. Symptoms and signs of heart failure:**  One or more of the following symptoms consistent with heart failure:  a. Worsening dyspnoea  b. Worsening orthopnoea  c. Paroxysmal nocturnal dyspnoea  d. Increasing fatigue/ worsening exercise tolerance  **AND**  Two or more of the following signs consistent with heart failure:  a. Rapid weight gain  b. Pulmonary oedema or rales  c. Elevated jugular venous pressure  d. Radiologic signs of heart failure  e. Peripheral oedema  f. Increasing abdominal distension or ascites  g. S_3_ gallop  h. Hepatojugular reflux  i. Elevated brain natriuretic peptide (BNP) or N-terminal pro-BNP (>most recent baseline)  **AND**  **2. Treatment**  Treatment with intravenous diuretics, intravenous vasodilators, intravenous inotropes, mechanical fluid removal (e.g., ultrafiltration or dialysis), or insertion of an intra-aortic balloon pump for hemodynamic compromise. Initiation of standing oral diuretics or intensification (doubling) of the maintenance diuretic dose will also qualify as treatment. |
| RELAX-AHF-2 | **Rehospitalization due to heart failure:**  Rehospitalization due to heart failure will be defined as an event that occurred primarily because of the documented presence of at least 2 of the following 3 criteria:   - Clinical manifestations of heart failure - Biomarker or radiographic evidence consistent with heart failure - Use of additional or increased pharmacologic or mechanical interventions directed at the treatment of heart failure   Clinical manifestations of heart failure including the following signs and symptoms: New or worsening   - dyspnoea - orthopnoea - paroxysmal nocturnal dyspnoea - oedema - pulmonary rales - jugular venous distension - new or worsening third heart sound or gallop rhythm - hypotension or cardiogenic shock not occurring in the context of an acute myocardial infarction or as the consequence of an arrhythmia, or - other clinical evidence of new or worsening heart failure, eg, weight gain, or confinement to bed predominantly due to heart failure symptoms   Biomarker results consistent with heart failure include documented increased or increasing levels of a natriuretic peptide (BNP or NTproBNP)  Radiographic evidence consistent with heart failure includes documented worsening pulmonary congestion or pulmonary oedema on chest X-ray or other generally recognized imaging pattern.  The use of additional or increased pharmacologic or mechanical interventions directed at the treatment of heart failure includes:   - Initiation of intravenous diuretic, inotropic, or vasodilator therapy - Significant addition or increase in oral heart failure therapy - Up-titration of intravenous therapy, if already on therapy - Initiation of mechanical or surgical intervention (mechanical circulatory or ventilatory support, heart transplantation or ventricular pacing to improve cardiac function), or the use of ultrafiltration, hemofiltration, or dialysis that is specifically directed at treatment of heart failure |
| **Diabetes and pre-diabetes** | |
| ALTITUDE | **Unplanned Hospitalization for Heart Failure (HF)**  Presentation to an acute care facility requiring an overnight hospitalization (change in calendar day) with an unexpected exacerbation of heart failure requiring treatment meeting the following criteria:  **1. Symptoms and signs of heart failure:**  One or more of the following symptoms consistent with heart failure:  a. Increasing dyspnoea on exertion  b. Worsening orthopnoea  c. Paroxysmal nocturnal dyspnoea  d. Increasing fatigue/ worsening exercise tolerance  **AND**  Two or more of the following signs consistent with heart failure:  a. Rapid weight gain  b. Pulmonary oedema or rales  c. Elevated jugular venous pressure  d. Radiologic signs of heart failure  e. Peripheral oedema  f. Increasing abdominal distension or ascites  g. S3 gallop  h. Hepatojugular reflux  i. Elevated brain natriuretic peptide (BNP) or N-terminal pro-BNP  **AND**  **2. Treatment**  Treatment with either intravenous diuretics, intravenous vasodilators, intravenous inotropes, mechanical fluid removal (e.g., ultrafiltration or dialysis), or insertion of an intra-aortic balloon pump for hemodynamic compromise. Initiation of standing oral diuretics or intensification (doubling) of the maintenance diuretic dose will also qualify. |
| NAVIGATOR | **CHF requiring hospitalization**  Development of the signs and symptoms of CHF not present at screening and requiring hospital management or previously documented CHF that worsens, requiring hospital management.  CHF is clinically manifested by one or more of the following features:  a. Dyspnoea on exertion in the absence of new pulmonary disease  b. Paroxysmal nocturnal dyspnoea (shortness of breath that awakens the patient from sleep)  c. Orthopnoea (sleeping on two or more pillows to facilitate breathing)  **AND** one or more of the following criteria:  a. Pulmonary rales >1/3 of the way up the lung fields present after coughing in the absence of chronic lung disease or respiratory infection.  b. Pulmonary oedema on chest x-ray in absence of high suspicion for non-cardiac origin  c. New use of oral/intravenous diuretics, intravenous inotropes, intravenous vasodilators, or adjustment of previous diuretic dose  d. Oxygen desaturation (<90%) with no evidence of acute or chronic lung disease  e. Jugular venous distention (JVD)  f. Bilateral pedal oedema  g. Cardiomegaly (cardiothoracic ratio ≥0.55)  h. Left ventricular ejection fraction ≤0.40 (new or presumably new)  i. Left ventricular fractional shortening <0.25  j. S_3_ gallop on auscultation  k. Elevated BNP level |

## **Table S3: Agreement rates between investigator-reported and CEC-adjudicated events by region**

| **Study** | **Region** | **CV death n (%)** | **HFH n (%)** |
| --- | --- | --- | --- |
| PARADIGM-HF | Asia/Pacific and Other | 259 (97.7) | 215 (66.6) |
|  | Central/Eastern Europe | 388 (97.5) | 388 (71.9) |
|  | Latin America (including Central America) | 205 (96.7) | 118 (51.5) |
|  | North America | 72 (93.5) | 132 (76.3) |
|  | Western Europe | 169 (94.4) | 237 (70.1) |
|  | **-Overall** | **1093 (96.6)** | **1090 (68.0)** |
| ATMOSPHERE | Asia/Pacific and Other | 333 (99.1) | 311 (64.9) |
|  | Central/Eastern Europe | 383 (97.2) | 273 (57.7) |
|  | Latin America (including Central America) | 246 (96.5) | 158 (62.2) |
|  | North America | 32 (97.0) | 42 (71.2) |
|  | Western Europe | 291 (94.2) | 344 62.8) |
|  | **-Overall** | **1285 (96.8)** | **1128 (62.2)** |
| PARAGON-HF | Asia/Pacific and Other | 51 (98.1) | 117 (62.9) |
|  | Central/Eastern Europe | 98 (86.7) | 212 (73.4) |
|  | Latin America (including Central America) | 18 (94.7) | 31 (58.5) |
|  | North America | 38 (92.7) | 160 (81.2) |
|  | Western Europe | 69 (89.6) | 217 (63.8) |
|  | **-Overall** | **274 (90.7)** | **737 (69.2)** |
| ASTRONAUT | Asia/Pacific and Other | 102 (99.0) | 92 (79.3) |
|  | Central/Eastern Europe | 74 (98.7) | 105 (77.8) |
|  | Latin America (including Central America) | 17 (100.0) | 34 (75.6) |
|  | North America | 5 (100.0) | 53 (88.3) |
|  | Western Europe | 51 (100.0) | 123 (80.4) |
|  | **-Overall** | **249 (99.2)** | **407 (80.0)** |
| RELAX-AHF-2 | Asia/Pacific and Other | 14 (100.0) | 31 (86.1) |
|  | Central/Eastern Europe | 256 (94.8) | 370 (89.6) |
|  | Latin America (including Central America) | 56 (91.8) | 91 (90.1) |
|  | North America | 33 (86.8) | 167 (86.5) |
|  | Western Europe | 142 (90.4) | 413 (86.9) |
|  | **-Overall** | **501 (92.8)** | **1072 (88.0)** |
| ALTITUDE | Asia/Pacific and Other | 140 (95.9) | 85 (53.1) |
|  | Central/Eastern Europe | 82 (90.1) | 77 (68.1) |
|  | Latin America (including Central America) | 73 (85.9) | 26 (51.0) |
|  | North America | 46 (92.0) | 78 (70.3) |
|  | Western Europe | 138 (89.0) | 153 (62.4) |
|  | **-Overall** | **479 (90.9)** | **419 (61.6)** |
| NAVIGATOR | Asia/Pacific and Other | 13 (81.3) | 8 (66.7) |
|  | Central/Eastern Europe | 39 (86.7) | 40 (67.8) |
|  | Latin America (including Central America) | 63 (85.1) | 30 (75.0) |
|  | North America | 41 (87.2) | 40 (72.7) |
|  | Western Europe | 55 (94.8) | 46 (78.0) |
|  | **-Overall** | **211 (87.9)** | **164 (72.9)** |

## **Figure S1: Log(ratio of hazard ratios) for main regions, with reference Asia/Pacific and Other**


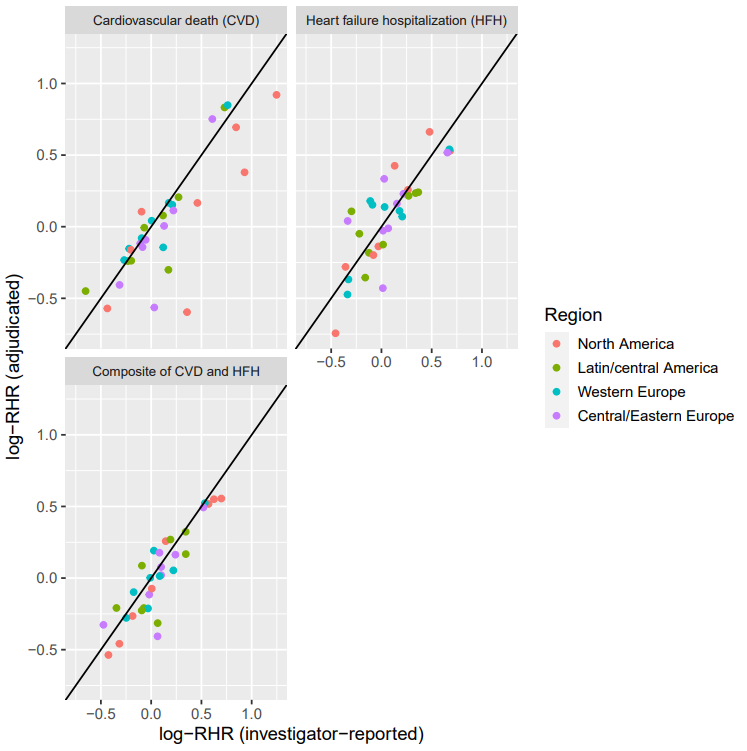


The circles reflect the log(RHR) for each treatment comparison and each region versus Asia/Pacific and Other as reference. The underlying Cox model is specified as h(t)=h_0_(t)*exp(beta_region_*region + beta_trt_*trt + beta_region_*trt*trt*region). Therefore, defining HR_1_=exp(beta_region_*region + beta_trt_*trt + beta_region*trt_*trt*region) and HR_2_=exp(beta_region_*region + beta_trt_*trt), we obtain HR_1_/HR_2_=exp(beta_region*trt_*trt*region)=Ratio of Hazard Ratios.
